# Activity-based chemical proteomics reveals caffeic acid ameliorates pentylenetetrazol-induced seizures by covalently targeting aconitate decarboxylase 1

**DOI:** 10.1186/s12964-024-01739-y

**Published:** 2025-02-03

**Authors:** Guanjun Li, Ling Huang, Di Gu, Peili Wang, Letai Yi, Wenhua Kuang, Ying Zhang, Junzhe Zhang, Dandan Liu, Qiaoli Shi, Huan Tang, Jichao Sun, Guohua Zeng, Xin Peng, Jigang Wang

**Affiliations:** 1 Department of Urology, Guangdong Provincial Clinical Research Center for Geriatrics, Shenzhen Clinical Research Center for Geriatric, Shenzhen People’s Hospital (The Second Clinical Medical College, Jinan University; The First Affiliated Hospital, Southern University of Science and Technology), Shenzhen, 518020 Guangdong China; 2 Guangdong Key Laboratory of Urology, The First Affiliated Hospital of Guangzhou Medical University, Guangzhou, 510230 Guangdong China; 3 Xiyuan Hospital, National Clinical Research Center for Chinese Medicine Cardiology, Academy of Chinese Medical Sciences, Beijing, China; 4 Inner Mongolia Medical University, 010000 Hohhot, Inner Mongolia China; 5 State Key Laboratory for Quality Ensurance and Sustainable Use of Dao-di Herbs, Artemisinin Research Center, Institute of Chinese Materia Medica, China Academy of Chinese Medical Sciences, 100700 Beijing, China; 6 Ningbo Municipal Hospital of TCM, Affiliated Hospital of Zhejiang Chinese Medical University, 315000 Ningbo, Zhejiang China; 7 State Key Laboratory of Antiviral Drugs, School of Pharmacy, Henan University, 475004 Kaifeng, China

**Keywords:** Phytomedicine, Natural products and herbs, Network pharmacology, Activity-based chemical proteomic

## Abstract

**Background:**

Epilepsy is a neurological disorder characterized by recurrent seizures, tightly associated with neuroinflammation. Activation of inflammatory cells and molecules in damaged nervous tissues plays a pivotal role in epilepsy. Caffeic acid, one of the most abundant polyphenols in coffee, has shown potent protective effects as a phytomedicine in various neurological disorders. However, the direct protein targets and exact molecular mechanisms of caffeic acid in epilepsy, remain largely elusive.

**Purpose:**

This study aimed to explore the protective effects of caffeic acid in epilepsy and elucidate its underlying mechanism.

**Methods:**

In this study, we established pentylenetetrazol-induced acute and kindling models of seizures. Additionally, a BV2 microglial cellular inflammation model was established by lipopolysaccharide stimulation. The potential direct protein targets of caffeic acid in BV2 cells were analyzed using an activity-based protein profiling (ABPP) with a caffeic acid probe. Various methods such as pull-down assay, immunofluorescence and cellular heat transfer assays were used for experimental validation. The anti-inflammatory effects of caffeic acid in LPS-activated BV2 cells was proved by knocking down the target protein.

**Results:**

Here, we found that caffeic acid exhibits antiepileptic effects in pentylenetetrazol-induced epilepsy mice and exerts anti-neuroinflammation effect in vivo and in vitro. Besides, we discovered that caffeic acid directly binds to aconitate decarboxylase 1 and influenced its enzymatic activity. Moreover, we indicated that caffeic acid exhibits anti-neuroinflammation effect through aconitate decarboxylase 1 mediated PERK-NF-κB pathway in vitro.

**Conclusion:**

In summary, this study elucidates, for the first time, the potential antiepileptic targets and mechanism of action of caffeic acid using the ABPP strategy. Our study provides evidence supporting the utilization of caffeic acid as a promising therapeutic agent for treating epilepsy and neuroinflammation-related disorders.

**Supplementary Information:**

The online version contains supplementary material available at 10.1186/s12964-024-01739-y.

## Highlights

1. Caffeic acid alleviates antiepileptic effects in pentylenetetrazol-induced epilepsy and exhibits obvious anti-neuroinflammatory effect.

2. Caffeic acid directly covalently binds to the cysteines of aconitate decarboxylase 1 (ACOD1).

3. Caffeic acid ameliorates neuroinflammation by inhibiting the ACOD1-PERK-NF-κB pathway.

**Supplementary Information:**

The online version contains supplementary material available at 10.1186/s12964-024-01739-y.

## Background

Epilepsy is a chronic neuroexcitability disorder characterized by excessive synchronization and abnormal discharges of neurons in the brain [1]. In addition, inflammatory processes, such as the generation of inflammatory factors and related molecules, have been noted to increases the risk of acquired epilepsy in humans and relevant animal models [2]. Numerous cytokines, including IL-1β and IL-6, have been shown to be associated with epilepsy [3, 4]. Disruption of glial-neuronal networks may play a crucial role in epilepsy, particularly by causing the activation of microglia [5]. Microglia are the primary immunological cells in the central nervous system (CNS), mediating neuroinflammatory responses [6, 7]. Persistent activation of microglia can exacerbate a broad range of chronic neuroinflammation, significantly increasing inflammatory cytokines such as interleukin-1 (IL-1) and interleukin-6 (IL-6), which can cause or exacerbate seizures [8, 9]. Anti-inflammatory treatments could serve as interventions and may represent promising strategies for the prevention and treatment of seizures and other associated neurological disorders.

Caffeic acid (CA) is a natural polyphenolic compound found in a variety of foods, including coffee and tea [10]. Numerous published studies have demonstrated the effects of CA on various neurological disorders [11], but few have specifically investigated its role in epilepsy. However, the specific target proteins and potent molecular mechanisms underlying CA’s antiepileptic effects remain unclear. Activity-based protein profiling (ABPP), a powerful method based on chemical proteomic strategies, has been utilized to characterize the protein targets of small bioactive molecules [12]. By using ABPP with liquid chromatography liquid chromatography-tandem mass spectrometry (LC-MS/MS) analysis, we have proved the direct targets of several natural compounds in different disease models before [13–15]. Here, we aimed to elucidate the role of CA in epilepsy and its associated neuroinflammatory responses, and to uncover the underlying mechanism using the ABPP strategy.

Aconitate decarboxylase 1 (ACOD1) is an enzyme that catalyzes the conversion of cis-itaconate to itaconate, originally identified as a gene induced by bacterial lipopolysaccharide (LPS), and is involved in the innate defense of mouse macrophages [16]. ACOD1 accumulation is reported to induce proinflammatory responses through itaconate-independent pathways. Aberrant expression of ACOD1 has been associated with inflammatory responses and the activation of cytokine storms, resulting in significant upregulation of cytokines [17, 18].

In this study, we demonstrated that a natural polyphenolic compound, CA, has a significant effect on pentylenetetrazol (PTZ)-induced seizures in mice, a commonly used model of epilepsy. Furthermore, our experiments elucidated the potential molecular mechanisms through ABPP. Collectively, these findings provide novel insights into the molecular mechanisms of CA in epilepsy.

## Materials and methods

### Animal experiments

Adult male C57BL/6J mice were housed in groups. In neuroscience research, it has been suggested by several studies that the estrous cycle of females may influence animal behavior [19, 20], therefore, male mice were utilized in this study. All animal experiments were approved by the Care and Use of Laboratory Animals Center of Shenzhen People’s Hospital (Approval No: AUP-230,725-LGJ-0287-01). Mice received DMSO or CA (10 mg/kg or 20 mg/kg) by intragastric administration daily for 7 days. Inflammation model mice received LPS (10 mg/kg) by injection.

### **PTZ-induced acute and kindling models of seizures**

Pentylenetetrazol (PTZ, P6500, Sigma, 55 mg/kg) was administered to induce acute seizures. The PTZ kindling model of epileptogenesis was utilized to investigate the effect of CA on seizure onset. Mice were administered 40 mg/kg of PTZ every other day to induce kindling, and received daily doses of DMSO or CA (10 mg/kg or 20 mg/kg). Seizure onset (seizure latency) was determined by the time elapsed from PTZ administration to the first observed seizure. Seizures were assessed using a 5-point seizure scale (seizure score): 0) absence of behavioral symptoms; (1) whisker twitching and/or facial and neck jerking; (2) sitting clonic seizures; (3) tonic-clonic seizures (abdominal recumbency); and (4) tonic-clonic seizures (lateral recumbency) or manic jumping. All behavioral experiments were conducted by an observer who kept the experimental conditions confidential in order to reduce biasness.

### Cell culture

The mouse microglia BV2 cell line was obtained from the Cell Resource Centre, Institute of Basic Medical Sciences, Chinese Academy of Medical Sciences (M011, China). BV2 cells were cultured in DMEM containing 10% fetal bovine serum (FBS), as well as 100 U/mL penicillin and streptomycin (PS). BV2 cells were maintained at 37 °C with 5% CO2 humidified atmosphere. Inflammatory BV2 cell model was induced by adding LPS (1 µg/ml) for 24 h.

### Detection of cell viability

To assess cell viability, the CCK-8 experiment was conducted following the protocols. BV2 cells were initially cultured in 96-well plates overnight, and treated with various doses of CA or CA-P for 24 h.

### Measurement of NO and inflammatory cytokines

BV2 cells were initially cultured with LPS overnight, followed by the addition of CA or CA-P. Subsequently, the nitric oxide (NO) in the culture supernatant of BV2 cells was assessed using Nitric Oxide Assay Kit (Biodee, DEM106), following the manufacturer’s protocol.

Besides, Elisa kits were used to test the levels of inflammatory cytokines including TNF-a (Biorigin, BN50578), IL-1β (Biorigin, BN50543) as well as IL-6 (Biorigin, BN50553) in hippocampus tissues, serum of mice and culture medium, according to previous reports [21]. Serum was isolated from the blood taken from eyeballs of mouse.

### Measurement of ACOD1

The Elisa kits were used to measure the content (Mlbio, YJ1050411) as well as enzymatic activity (Mlbio, YJ1050412) of ACOD1 in the cell supernatants of BV2 cells according to the manufacturer’s instructions.

### Cellular Imaging

Fluorescence imaging experiments were conducted to show the localization of ACOD1 and probe. Specific experimental methods were based on previous articles reported [22]. LPS-induced BV2 cells were incubated in glass bottom dishes. After treatment with or without of CA for 3 h, CA-P was added for 1.5 h, and cells were then washed with PBS. Subsequently, cells were fixed with 4% paraformaldehyde for 15–20 min and permeabilized in 0.2% Triton X-100 for 10 min at room temperature (RT). Cells were added with click reactions (TBTA (100 µmol/L), TCEP (1 mmol/L), CuSO4 (1 mmol/L) and biotin-azide (50 µmol/L) for 1 h at RT, followed by incubation with anti-ACOD1 (Thermo Fisher, PA5-102893, 1:200) at 4℃ overnight. Cells were then incubated with secondary fluorescent antibody (Alexa Fluor 488, Abcam, ab150077) (1:500) for 1 h. After that, Hoechst (1:5000) were added at RT for 10 min. Images were captured under confocal microscope. The intensity of fluorescence was analyzed using imageJ software based on previous report and the official website of imageJ [23]. We evaluate the intensity and remove background fluorescence by using the setting “Threshold” and keeping the threshold setting the same in all the images to be compared.

### Protein labeling

The detailed methods were mentioned as before [24]. LPS-induced BV2 cells were treated with CA-P or DMSO for 3 h, and cellular proteins were extracted with RIPA buffer (Solarbio, R0020). After that, proteins were treated with click chemistry reactions for 1 h at RT. Labeled protein was precipitated using prechilled acetone at -20 °C. After that, samples were resolubilized in loading buffer and fractionated by 10% SDS-PAGE, and then visualized through Azure Sapphire.

In competitive protein labeling experiments, cell samples were treated with CA and then CA-P. The following click chemistry and electrophoresis were the same as above.

Similar to the experiment described above, recombinant purified ACOD1 proteins were treated with CA for 3 h at RT and CA-P for 1.5 h, and following experiments were the same as above.

### Pull down Western blot and LC-MS/MS based targets identification

Cell lysates were extracted according to a previous report [25]. BV2 cells were incubated with CA or equal volume of DMSO for 3 h, all samples were sedimented with acetone and then re-dissolved in 0.1% SDS, treating with NeutrAvidin beads for 4 h at RT. The beads were washed with 1% SDS, 6 mol/L urea and 1×PBS for three times, respectively. To perform the digestion, beads enriched with proteins were excised, minced, reduced and alkylated with dithiothreitol (DTT)as well as iodoacetaamide (IAA), then digested using trypsin overnight at 37℃. The obtained peptides were collected and demineralized on C18 columns and then registered with TMT. Finally, samples were submitted. The pull down-western blot assay was performed to validate the target proteines by WB assay as previously described [26].

### GO and KEGG enrichment analysis

For data analysis, the abundance changes in the control, CA-P or compete groups in the MS were used to distinguish differential proteins according to absolute fold change ≥ 2 or 5 at the same time p-value < 0.05 [27]. Then selected proteins were exhibited through scatter plots and volcano plots analysis via bioladder website. In addition, Veen and GO enrichment analyses were carried out on the selected proteins by DAVID^+^ version.

### Purification of recombinant ACOD1

Wide type mouse ACOD1 gene was cloned into pet30a vector and transformed into E. colistrain B21. Plates were coated to obtain monoclonal colonies which were reproduced by shaking at 25 °C at 200 rpm and then induced overnight at 17 °C with isopropyl-D-1-thiogalactopyranoside (IPTG). Then the samples were collected, lysed, and centrifuged for 30 min. Samples were incubated with Nickel column with a His tag for 2 h. After that, samples were washed with different concentrations of imidazole (10, 20, 50, 100, 200 and 300mM). The imidazole concentration of protein elution was determined by SDS-PAGE, and then the extract was concentrated by ultrafiltration to obtain ACOD1, which was quantified by BCA and diluted to 1 mg/ml, and stored at -80 °C.

### Western blotting analysis

According to previous reports [28], total proteins in BV2 cells were extracted, separated by 10% SDS-PAGE. Proteins were incubated with antibodies against TNF-α (Proteintech, 17590-1-AP, 1:2000), IL-1β (Proteintech, 26048-1-AP, 1:1000), ACOD1 (Proteintech, 28436-1-AP, 1:2000), p-PERK (Proteintech, 29546-1-AP, 1:1000), NF-κB p65 (Proteintech, 10745-1-AP, 1:2000) and β-actin (Proteintech, 20536-1-AP, 1:5000), followed by the corresponding secondary antibodies (goat anti-rabbit, 1:4000 and goat anti-mouse, 1:4000). These bands were visualized using enzyme-linked chemiluminescence and analyzed through Image J software.

### Cellular thermal shift assay (CETSA)

As previously published protocols with minor modifications [29], the cell lysate was collected. The protein samples were added with DMSO or CA, and then divided into equal parts and heated in designated temperatures (37–72 °C). for further WB experiment.

### Molecular docking

The crystal structure of ACOD1 was retrieved from Protein Data Bank. We use Autodock software to perform dehydration and hydrogenation of proteins, and Pyrx-0.8 to do molecular docking as well as map [30].

### RNA interference and transfection

The detailed experimental procedure is similar as previously published protocols [31]. SiRNA was transfected into BV2 cells for 48 h for further WB analysis. Complementary oligonucleotides sequences of ACOD1 SiRNAs (CCGTCATTCTTTCCAGTAT) were synthesized by Ribobio.

### Statistical analysis

All data were presented as mean ± SEM values for at least three independent experiments. Statistical analysis was carried out by one-way ANOVA or two-way ANOVA in multiple groups. The density of WB bands was quantified through Image J software. All statistical data were calculated in GraphPad Prism 9.0 software.

## Results

### CA protects mice from neuroinflammation and epilepsy

**Fig. 1:**
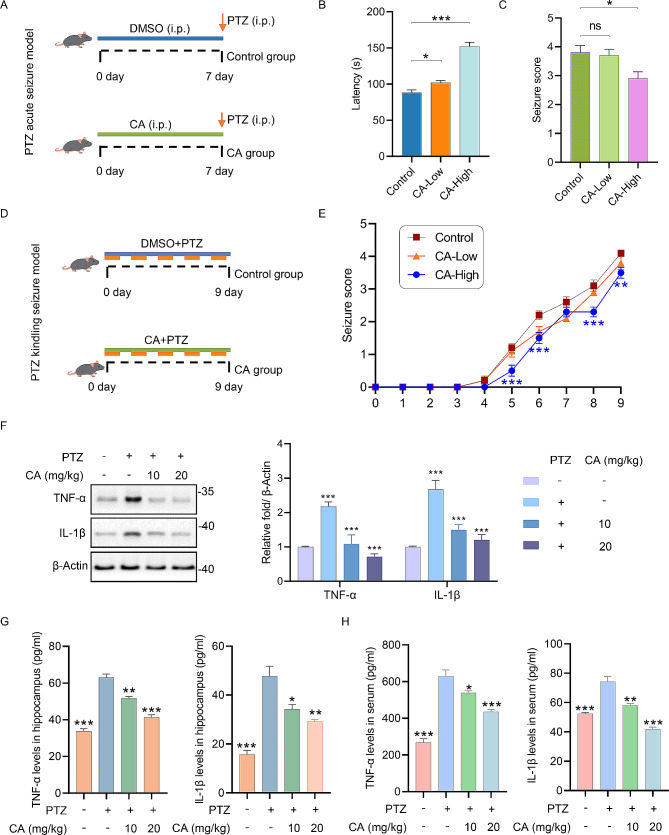
**CA suppresses neuroinflammation and epilepsy*in vivo.*** (A) Timeline of the PTZ acute epilepsy modeling. (B-C) Acute model of epilepsy. Statistical analysis of latency (B) and seizure scores (C) for acute response to PTZ in control and CA (10 mg/kg or 20 mg/kg)-treated mice (*n* = 10). (D) Timeline of the PTZ kindling seizure model. (E) Statistical analysis of seizure scores in PTZ kindling seizure mice (*n* = 10). (F) The expression levels and densitometry analysis of TNF-α and IL-1β in the hippocampus of PTZ-induced mice, *n* = 3. (G-H) The release of inflammatory factors in hippocampus and serum of PTZ-induced mice, *n* = 3. ns, not significant, *p < 0.05, **p < 0.01, ***p < 0.001 vs. control group in B-C and E. *p < 0.05, **p < 0.01, ***p < 0.001 vs. PTZ-induced group in F-H.

First, we investigated the anti-epileptic and anti-neuroinflammation effects of CA *in vivo.* Initially, we evaluated the protective effects of CA in a pentylenetetrazol (PTZ)-induced acute epilepsy model (Fig. 1A). Administration of CA per day for 7 days prior to PTZ administration markedly reduced seizure severity of mice. The response to PTZ was delayed in CA-treated mice (Fig. 1B), and the average seizure scores of PTZ-challenged mice in the CA group were lower than those in the control group (Fig. 1C). Furthermore, to investigate whether CA impacts epileptogenesis, mice were given repeated injections of 40 mg/kg PTZ to conduct the PTZ kindling seizure model (Fig. 1D). In the control group, PTZ increased the severity of seizures after 9 injections, whereas concurrent administration of 20 mg/kg CA significantly reduced the mean seizure score (Fig. 1E). The original list of seizure latency and score of each mouse, along with a summary, were provided in Additional file 2_Table S1. Next, to further explore whether the inflammation was caused in PTZ-induced seizures model and examine the effects of CA on pro-inflammatory cytokines in PTZ-induced kindling model mice, western blotting was performed to examine the levels of TNF-α and IL-1β in the hippocampus of mice. The levels of TNF-α and IL-1β were significantly elevated in the PTZ-induced seizures model compared to the control group, which were reduced after the treatment of CA (Fig. 1F). Similarly, the Elisa results also showed that the levels of TNF-α and IL-1β both in the hippocampus and serum of mice were significantly increased in the PTZ group, and CA treatment significantly inhibited the levels of TNF-α and IL-1β (Fig. 1G-H). Additionally, the LPS-induced (10 mg/kg) inflammation model in mice is widely used to study inflammation [32]. Our results showed that CA significantly decreased the levels of inflammatory cytokines induced by LPS in the hippocampus and serum (Figure S1).

### CA inhibits inflammatory cytokines in LPS-induced BV2 cells

**Fig. 2:**
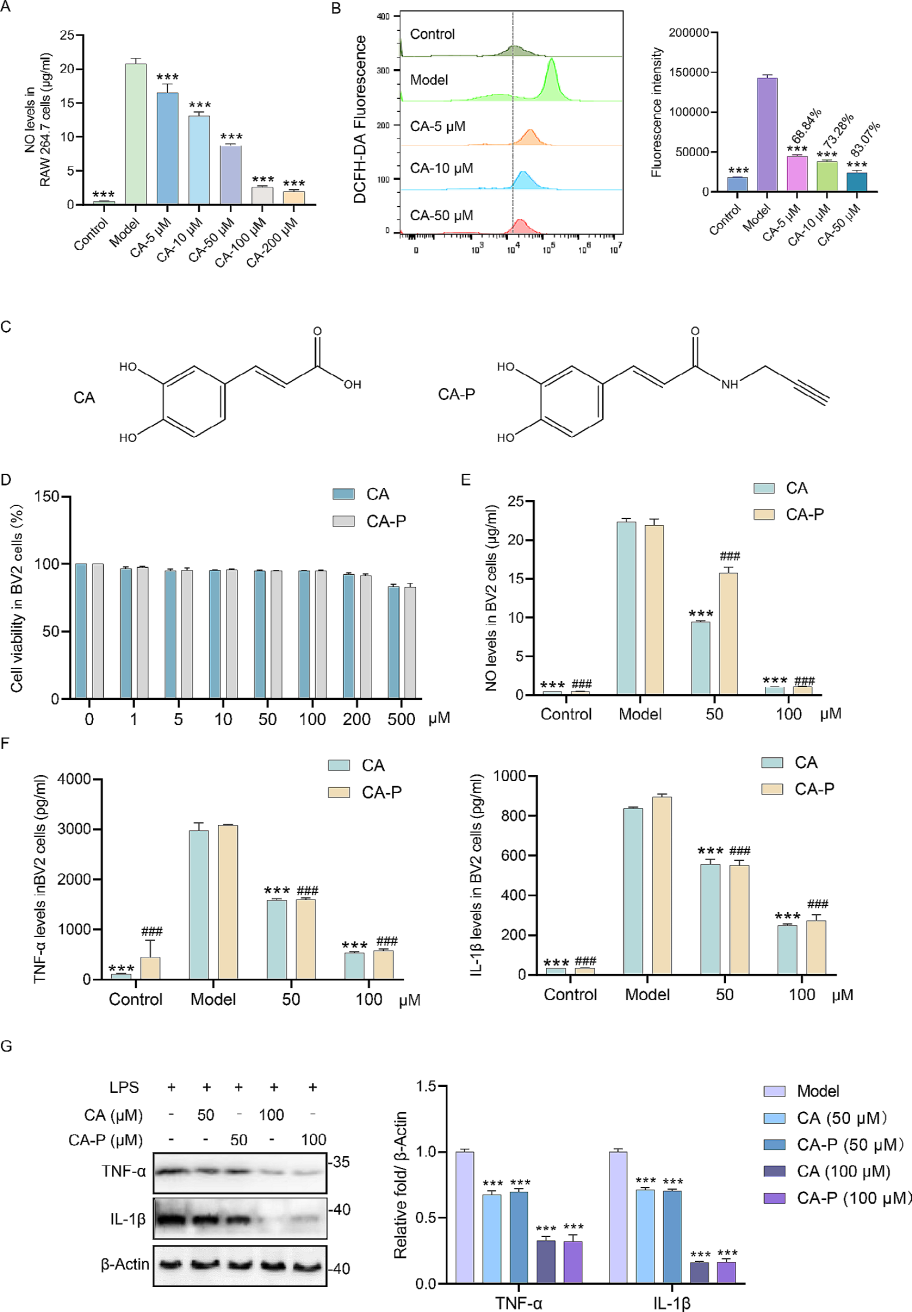
**Synthesis and bioactivity of CA-P in BV2 cells**. (A) The release levels of NO in BV2 cells with different concentrations of CA, *n* = 6. (B) The levels and quantitative statistical result of DCFH-DA measured by flow cytometry in BV2 cells with or without CA. Reduction (%) = 100%-(Fluorescence intensity of model group / Fluorescence intensity in CA-treated group) (%), and 68.84%, 73.28%, 83.07% means the average reduction of DCFH-DA. (C) Chemical structures of CA and CA-P. (D) Cell viability of CA and CA-P in LPS-treated BV2 cells, *n* = 3. (E-F) The release of NO and inflammatory factors in BV2 cells with treatment of CA or CA-P, *n* = 3. (G) The expression levels and densitometry analysis of TNF-α as well as IL-1β in BV2 cells, *n* = 3. ***p < 0.001, ^###^*p* < 0.001 compared with model group

Continuously, we explored the anti-neuroinflammation effects of CA in vitro. Activated microglia could aggravate brain inflammation, leading to subsequent injury that could cause or worsen seizures. Therefore, we attempted to assess the neuroprotective actions of CA using BV2 microglia [33]. BV2 cells, which retain a wide range of morphological, phenotypic, and functional characteristics of microglia, is a immortalized mouse-derived microglial cell line [34]. In this study, BV2 cells were stimulated by LPS to establish an in vitro model of neuroinflammatory. The nitric oxide (NO) release levels of BV2 cells significantly decreased after treatment with CA (Fig. 2A). Extensive research has noted that reactive oxygen species (ROS) has an influential role in the progression of inflammation-related diseases [35, 36]. We further evaluated the ROS levels, and the flow cytometry results showed that CA inhibited the release of ROS in a dose dependent manner (Fig. 2B). Collectively, our results suggested that CA has the ability of anti-neuroinflammatory in vitro.

### Synthesis and bioactivity of the CA-probe (CA-P)

Given the apparent anti-neuroinflammatory activity of CA, exploring the target proteins and specific molecular mechanisms of CA is of great interest. Subsequently, we synthesized a CA-probe (CA-P) (Fig. 2C) and examined its biological activity. The cytotoxicity of CA and CA-P in BV2 cells was assessed using CCK-8 assay. CA-P displayed similar effect to CA on cell viability (Fig. 2D). Additionally, to further confirm that CA-P has similar anti-neuroinflammatory bioactivity to CA, the release of NO and inflammatory factors from BV2 cells was also examined. Results indicated that there were no obvious differences between CA and CA-P (Fig. 2E-F). In addition, Western Blot results indicated that CA as well as CA-P markedly reduced the expression levels of TNF-α and IL-1β (Fig. 2G). Taken all together, our results demonstrated that CA-P displays similar anti-neuroinflammation effects to CA, suggesting that CA-P possesses similar bioactivity with the parent molecule.

### Identification of CA targets via ABPP-based chemical proteomics

**Fig. 3:**
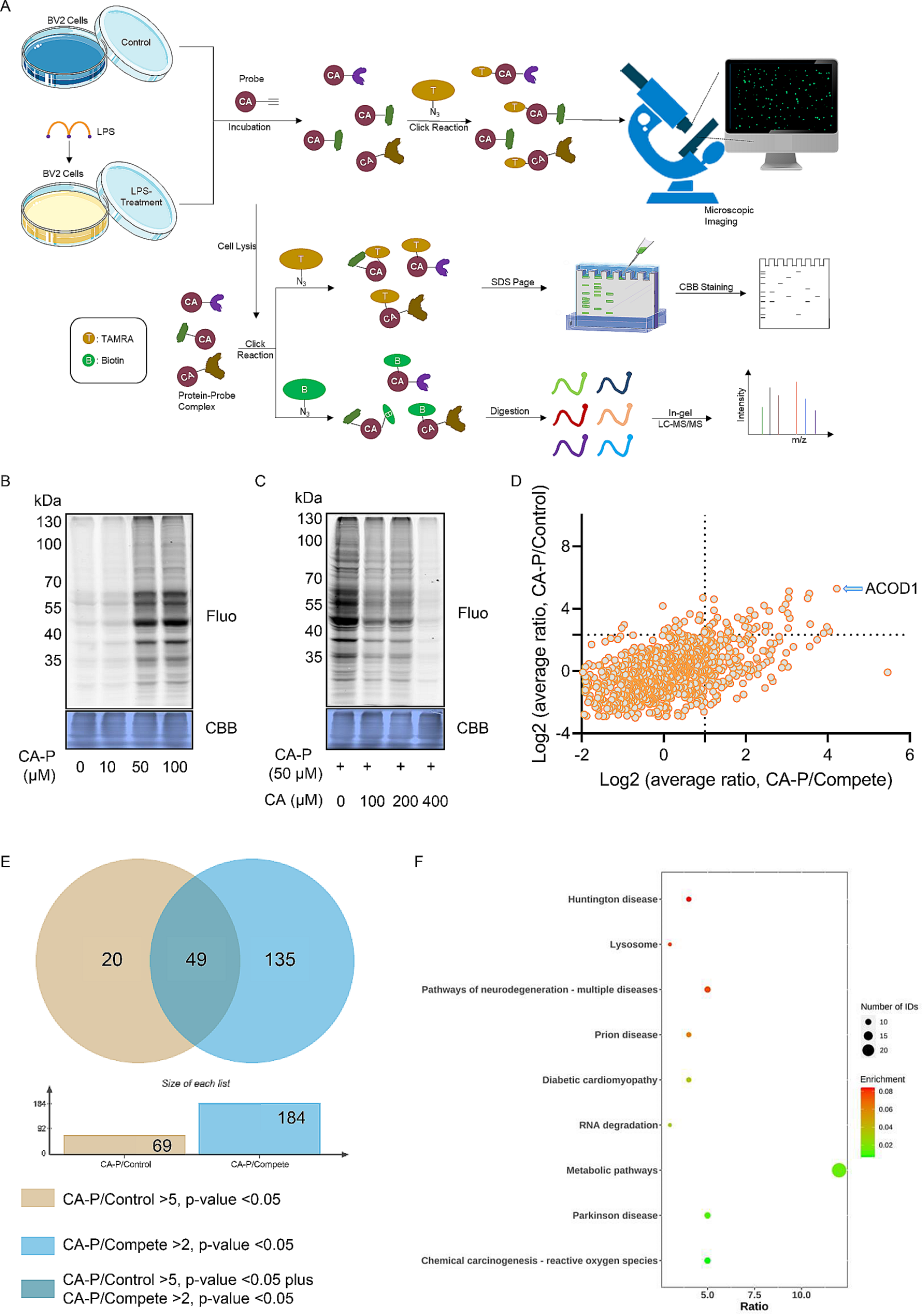
**Analysis and determination of the CA target proteins in LPS-induced BV2 cells.** (A) ABPP integral worksheet for analyzing potential CA targets. (B) Protein labeled with CA-P in LPS-induced BV2 cells. (C) Competition for proteins tagged with CA-P and CA in LPS-treated BV2 cells. (D) Scatter plot depicting the differential enrichment of proteins. (E) Venn digraph displaying the overlap of target proteins significantly enriched. (F) Scatter diagram for differential target proteins profiles captured. Fluo, fluorescence; CBB, Coomassie blue

To understand how CA exerts its anti-neuroinflammatory effects, we conducted activity-based protein profiling (ABPP) to identify its potential targets (Fig. 3A). Three groups were prepared as follows: the control group consisted of BV2 cells incubated with vehicles (DMSO); the Probe group involved BV2 cells treated with CA-P; and the competition group comprised BV2 cells cultured with CA-P and CA. BV2 cells were incubated with CA-P or DMSO, and multiple bands representing potential targets were seen in the CA-P lanes compared to the control. The labeling effect was dose-dependent, and a suitable level of labeling was observed with 50 µM CA-P (Fig. 3B), which was chosen for further experiments. Additionally, the labeling signal intensity weakened dose-dependently with the addition of CA, and the labeling profiles disappeared with a pretreatment of 400 µM CA, implying that excessive CA competes with CA-P for target labeling (Fig. 3C).

Next, we employed a chemical proteomics method to identify the targets of CA. Probe-labeled proteins were purified using streptavidin and digested on beads. We then performed tandem mass tag quantitation and characterized the peptide fragments via mass spectrometry (MS). The enriched proteins were identified and visualized using corresponding scatter plots and volcano plots (Fig. 3D, Figure S2). Overlapping proteins between the CA-P/Control group and the CA-P/Competition group were selected to eliminate false-positive results. We identified 69 proteins with a fold change of CA-P/Control > 5 and 184 proteins with a fold change of CA-P/Competition > 2, and finally selected 49 overlapping proteins as the potential targets (Fig. 3E). The original list of proteins from MS was shown in Additional file 3_Table S2. Additionally, Kyoto Encyclopedia of Genes and Genomes (KEGG) analysis indicated that the CA targets were primarily enriched in metabolic pathways (Fig. 3F).

Among these proteins, aconitate decarboxylase 1 (ACOD1), also known as immune-responsive gene 1 (IRG1) [37], was highlighted as the top hit from the candidates (top1 in fold change of both CA-P/Control group and CA-P/Compete group). Therefore, we focused on ACOD1 in our subsequent target analysis to explore whether ACOD1 is one of the key target proteins of CA and its underlying mechanism.

### CA directly targets ACOD1 in LPS-induced BV2 cells

**Fig. 4:**
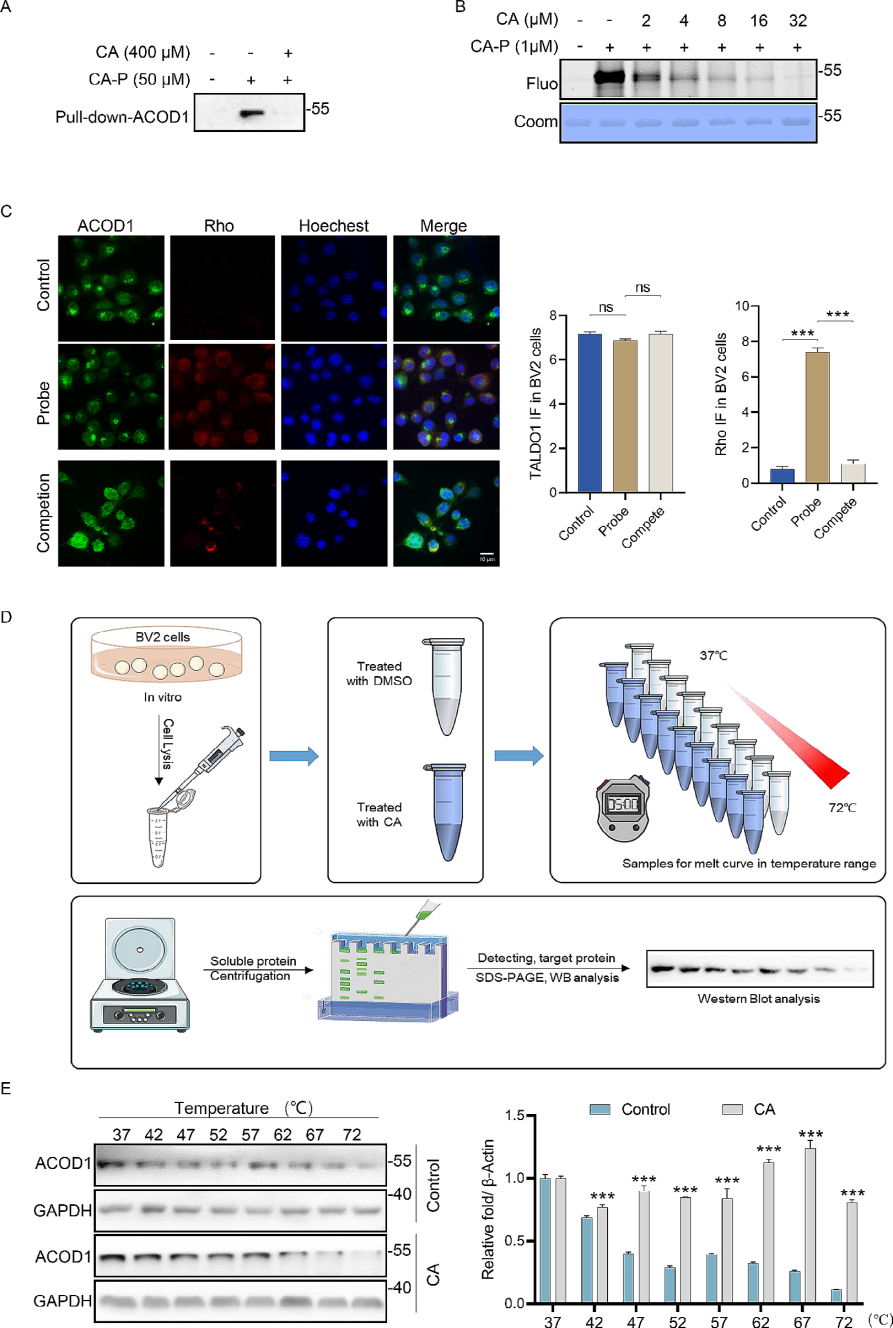
**Validation of the interaction between ACOD1 and CA.** (A) CA-P pull-down experiment in LPS-induced BV2 cells followed by western blot. (B) CA-P labeling of recombinant ACOD1, *n* = 3, ns, not significant, *** *p* < 0.001. (C) Immunofluorescence staining and statistical analysis in LPS-treated BV2 cells with antibodies against ACOD1 (green) and TAMRA click CA-P (red), scale bar = 10 μm. Control: DMSO; Probe: CA-P (50 µM), Competion, CA (400 µM) and CA-P (50 µM). (D) Scheme of CETSA-WB experiment. (E) CETSA assay and density analysis of protein levels in control and CA (400 µM)-treated LPS-induced BV2 cells, *n* = 3, **p < 0.01, *** *p* < 0.001 vs. control group. CETSA, the Cellular Thermal Shift Assay

To support the above conclusion, a pull-down western blotting experiment was performed. CA-P successfully pulled down ACOD1 after rigorous washing conditions and excess CA efficiently competed away the binding of CA-P to ACOD1 (Fig. 4A). Furthermore, recombinant ACOD1 protein was pre-treated with various doses of CA before the addition of CA-P, and CA competitively inhibited the binding of CA-P to the protein in a dose-dependent manner, indicating that CA covalently modified ACOD1 (Fig. 4B). Subsequently, immunofluorescence staining was conducted using an antibody against ACOD1 (green) and CA-P labeled with a red fluorescence dye. The results indicated co-localization of ACOD1 with CA-P, and the fluorescence signal from CA-P was attenuated by excess CA (Fig. 4C). Collectively, these results were in agreement with the LC-MS/MS findings. Next, we constructed cellular thermal shift assay (CETSA) followed with western blot experiment to assess the thermostable character of the targets (Fig. 4D). Proteins extracts from BV2 cell lysates were incubated with CA or DMSO, and then analyzed as reported previously [38]. The results displayed the heat stability of ACOD1 in the CA-treated group was improved as compared to the control group (Fig. 4E).

### CA covalently binds to ACOD1 and inhibits its enzymatic activity

**Fig. 5:**
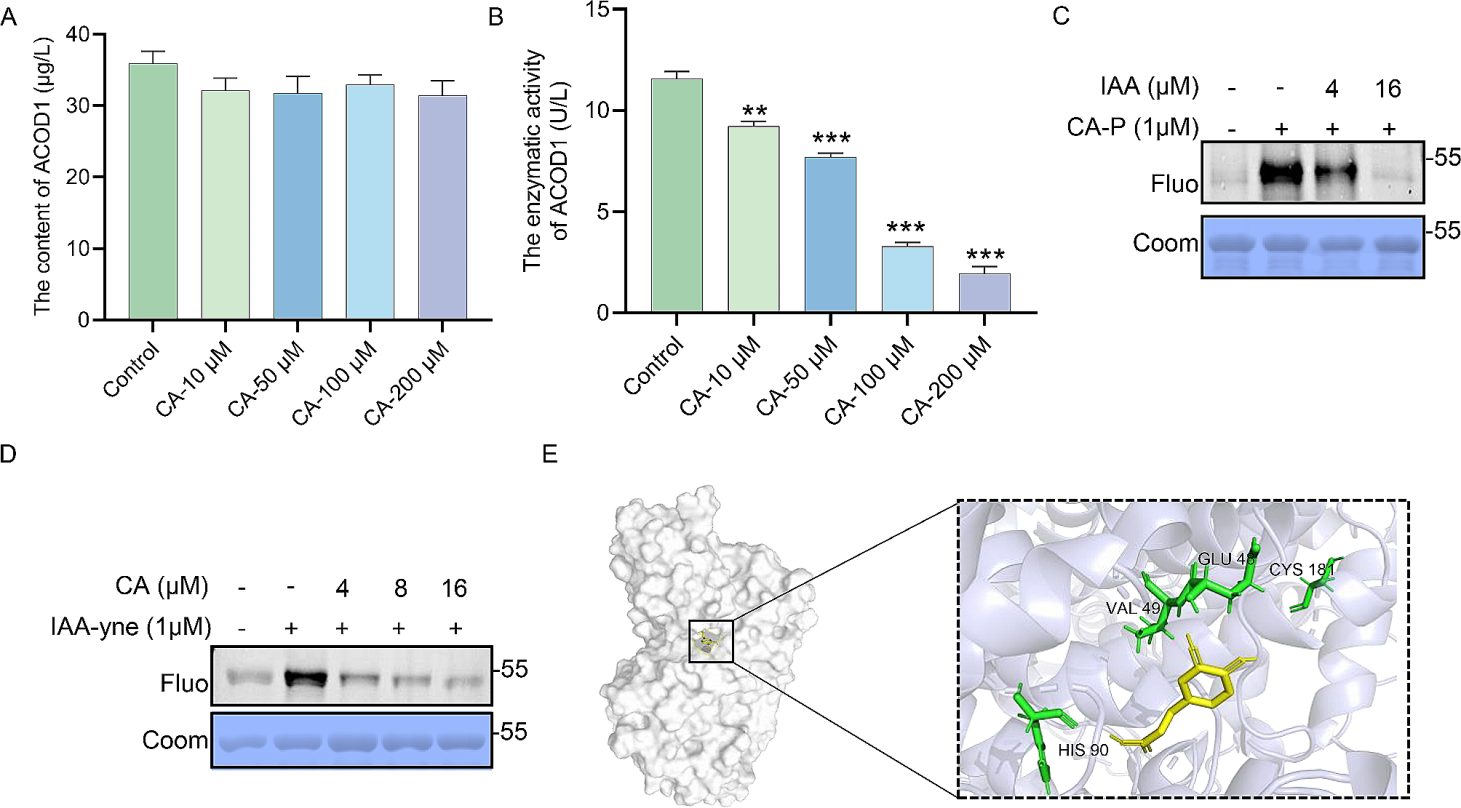
**CA covalently binds to ACOD1 and effects its enzymatic activity.** (A-B) The content (A) and enzymatic activities (B) of ACOD1 in BV2 cells after treated with different dose of CA, *n* = 5. (C) Labeling of recombinant ACOD1 with CA-P and IAA competitor. (D) Labeling of recombinant ACOD1 with IAA-yne and CA competitor. (E) Molecular docking model of CA with ACOD1 visualized by molecular docking. IAA, Iodoacetamide; IAA-yne, iodoacetamide-alkyne. **p < 0.01, ***p < 0.001 vs. control group

Since ACOD1 is an enzyme, we then investigated whether CA influences the content and enzymatic activity of ACOD1. The results indicated that CA inhibited the enzymatic activity of ACOD1 in a dose-dependent manner, but did not affect its content (Fig. 5A-B). Besides, considering that cysteine residues play an essential role in protein conformation [39] and CA contains electrophilic α,β-unsaturated carbonyl groups which may form Michael addition reactions with cysteine residues [40], we measured the binding of CA to cysteine residues of ACOD1. As expected, excess iodoacetamide (IAA, a reactive cysteine alkylating reagent) significantly competed with CA-P for labeling of recombinant protein ACOD1 (Fig. 5C). Besides, CA exhibited effective competition for the labeling of recombinant protein ACOD1 by IAA-yne (IAA bound to the alkyne moiety) dose-dependently (Fig. 5D). Overall, these findings indicated that the cysteine residues of ACOD1 could be occupied by CA. Furthermore, molecular docking analysis predicted that CA might interact with different residue sites of ACOD1, including Cys181 (Fig. 5E). This suggested that the Cys181 residue of ACOD1 may be covalent modified by CA.

### CA exhibited anti-neuroinflammation via ACOD1-PERK-NF-ΚB pathway

The Nuclear Factor kappa B (NF-κB) transcription factor plays a significant role in the regulation of various biological processes, including development, inflammation, and innate and adaptive immune responses [41]. It is reported that aberrant NF-κB activity is connected with inflammatory diseases [42]. Protein kinase RNA-like ER kinase (PERK), which is activated by a phosphorylated state (p-PERK) and thus induces the phosphorylation of NF-κB (NF-κB p65), may respond to the accumulation of misfolded proteins and inflammation, and specifically control inflammation [43].

**Fig. 6:**
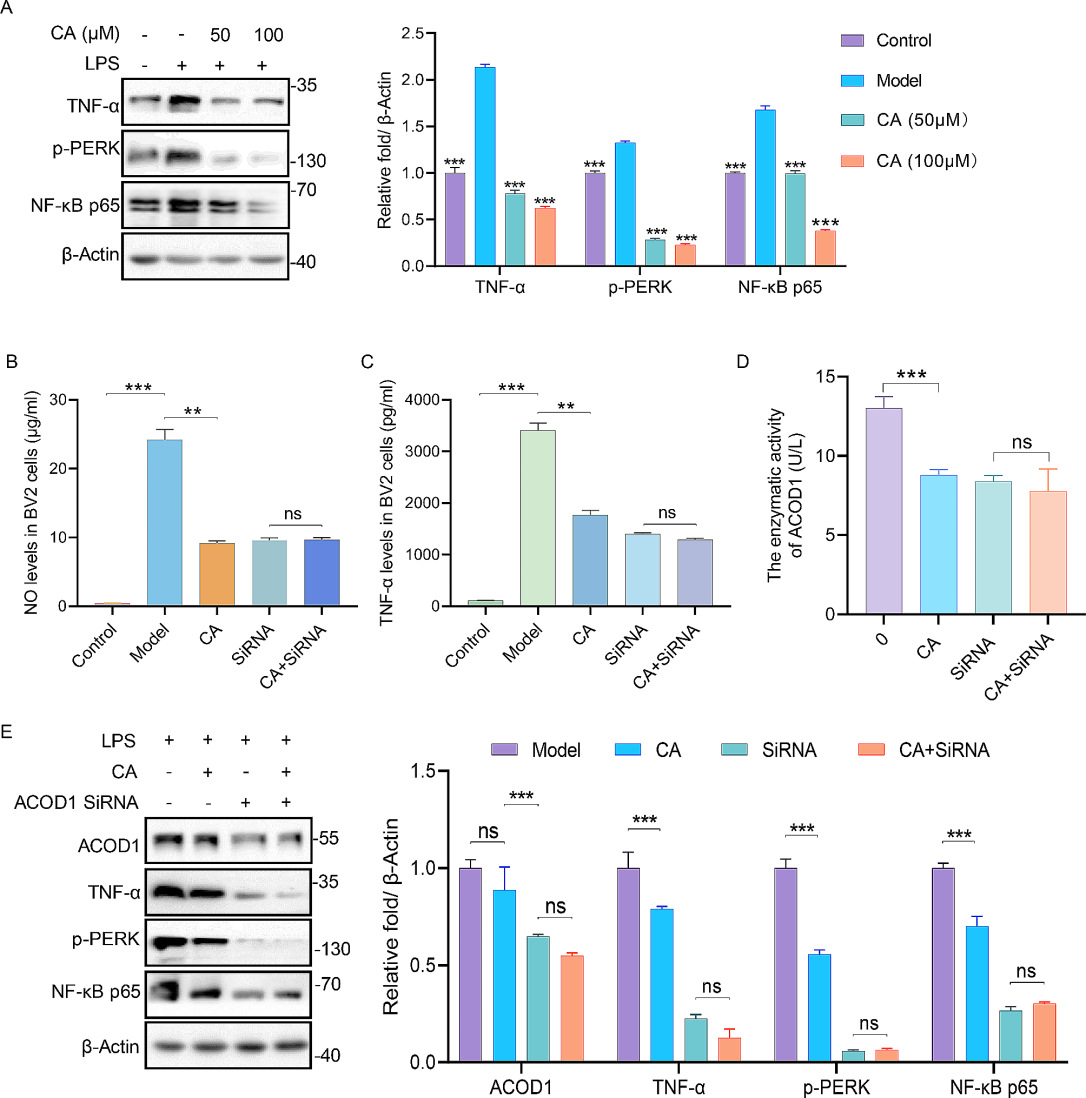
**CA suppressed neuroinflammation through NF-κB pathway.** (A) The expression levels and densitometry analysis of proteins in BV2 cells after treated with or without CA for 48 h, *n* = 3, ***p < 0.001 vs. model group. (B) The levels of NO in BV2 cells with CA (100 µM) and SiRNA against ACOD1, *n* = 5. (C) The levels of TNF-α in BV2 cells with CA (100 µM) and ACOD1 SiRNA, *n* = 5. (D) Elisa assay of enzymatic activity in BV2 cells after treated with CA (100 µM) and ACOD1 SiRNA, *n* = 5. (E) Expression and densitometry analysis of ACOD1, TNF-α, p-PERK as well as NF-κB-p65 in LPS-treated BV2 cells after transfected with a plasmid of ACOD1 SiRNA and treated with CA (100 µM) for 48 h, *n* = 3. ns, not significant, **p < 0.01, ***p < 0.001 in B-E.

We hypothesized that the anti-neuroinflammatory activity of CA is associated with the PERK-NF-κB pathway. CA dose-dependently abolished the increased expression of TNF-α, p-PERK and NF‐κB p65 in LPS-induced BV2 cells (Fig. 6A). Additionally, small interfering RNA (SiRNA) against ACOD1 dramatically abolished the ability of CA on reducing the levels of NO and inflammatory cytokines in LPS-induced BV2 cells (Fig. 6B-C, Figure S3), suggesting that ACOD1 is required for CA to exert its anti-neuroinflammatory effects. Furthermore, co-treatment with CA and ACOD1 SiRNA significantly diminished CA’s ability to inhibit ACOD1 enzymatic activity (Fig. 6D). Moreover, consistent with the above results, WB results also showed that by silencing ACOD1 in LPS-mediated BV2 cells, CA was not able to induce the reduction of TNF-α, p-PERK, and p-NF-κB p65 (Fig. 6E). In summary, our results confirmed that CA exhibited anti-neuroinflammation via ACOD1 mediated PERK-NF-ΚB pathway.

## Discussion

CA is pharmacologically active and can be used to treat a wide range of neurological disorders, which may be a new lead compound in drug discovery research [44]. To the best of our knowledge, this is the first time that CA has been elucidated to have a significant antiepileptic effect in a PTZ-mediated epilepsy model, which enriches the investigation of CA in neurological disorders. In addition, we identified that CA could resist neuroinflammation effect, which has been determined as a promoter of epileptogenesis. This is also consistent with previous findings that CA plays an essential role in neuroinflammatory processes [45]. CA is a promising anti-neuroinflammatory agent that holds promise for the treatment of a wider range of neurological disorders.

Our results demonstrated that CA treatment delayed seizure onset and seizure degree in PTZ-induced acute epilepsy model mice and retarded the epileptogenesis process in PTZ-mediated kindling epilepsy model mice. Numerous studies have examined the roles of neuroinflammation in epilepsy [46, 47]. Epilepsy is known to cause microglia activation, which in turn exacerbates brain inflammation and exacerbates seizures [48]. Besides, it is reported that glial-neuronal network in epileptogenes is a key medium for proper brain functions, which is destroyed in epileptic brains [5]. Here, we found that CA reduces the generation of inflammatory cytokines both in mice and LPS-activated BV2 microglia cells, which is consistent with previous studies. Besides, the primary microglia cells from PTZ-induced acute epileptic mice need to be further researched subsequently.

Leveraging a powerful chemoproteomic method, ABPP, we identified ACOD1 as a major covalent target of CA. This finding was supported by a series of thorough validations, including pulldown assays and immunofluorescence techniques. ACOD1 accumulation has been reported to activate a cytokine storm in LPS-activated cells [49]. In addition, we demonstrated for the first time that CA suppressed neuroinflammation by inhibiting the biological function of ACOD1 and ACOD1-mediated activation of PERK-NF-κB inflammation signaling pathway, suggesting that the inhibition of ACOD1 enzymatic activity could be regarded as a therapeutic strategy for neuroinflammatory associated disorders including epilepsy. In addition, caffeic acid has been reported to have systemic anti-inflammatory effects [50], but whether CA operates directly in the brain or produces a central effect to provide an anti-neuroinflammatory effect needs to be further investigated.

Furthermore, we recognized several limitations in our study that require further investigation. Firstly, we tested the antiepileptic activity of CA using only PTZ-induced epilepsy models, however, other epilepsy models are needed in subsequent studies. Additionally, we used male mice in this topic, maybe female mice may also be considered for inclusion in future experimental designs. Besides, it is noting that the potential mechanisms of CA worth to be further studied as the mechanism of CA may be multi-targeted. Moreover, CA (20 mg/kg) produced better antiepileptic effects, but whether long-term use of CA is safe still warrants further studies, as it is also critical to assess their safety for clinical applications and develop them into clinical drugs [51, 52]. In addition, although the experimental animal and cellular models were used to illustrate the antiepileptic effect of CA, additional clinical studies and rigorous clinical investigations should be carried out through human studies.

## Conclusion

**Fig. 7:**
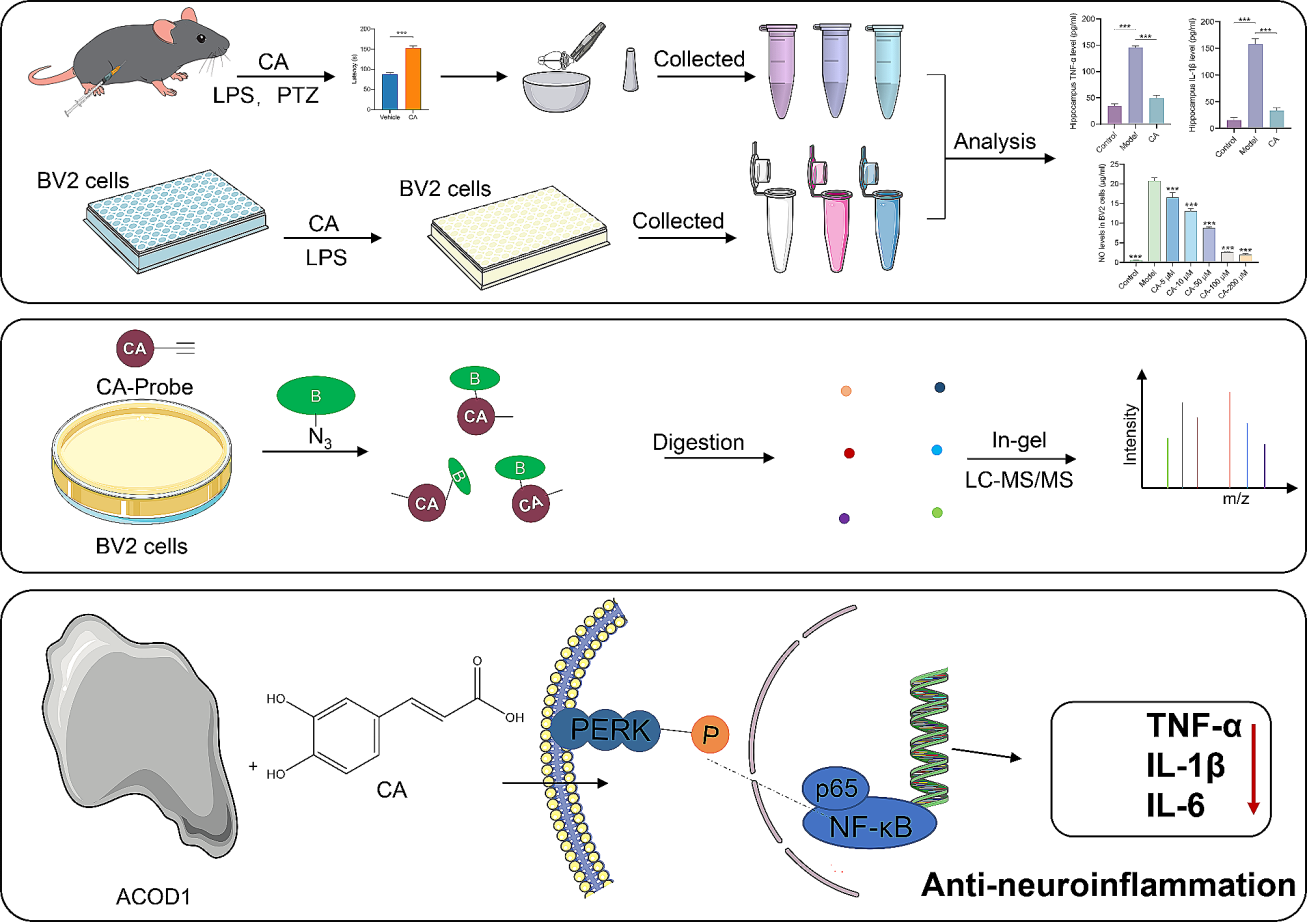
**Illustration of this study.** CA ameliorate PTZ-induced seizures and showed anti-neuroinflammation effect in vivo and in vitro. Besides, ABPP strategy was used to identify the direct target of CA. Moreover, CA exhibited anti-neuroinflammation via ACOD1 mediated PERK-NF-κB pathway

In summary, our findings suggested that CA could alleviate antiepileptic effects in PTZ-induced acute and kindling epilepsy model mice, and could reduce the production of LPS-induced inflammatory cytokines both in vivo and in vitro. Furthermore, we identified that CA directly targets ACOD1 through ABPP and inhibits its enzymatic activity. Our mechanistic study further revealed that CA dramatically inhibits neuroinflammation through the ACOD1 mediated PERK-NF-κB pathway (Fig. 7). Therefore, this work provides a new perspective that CA can be used as a novel therapeutic agent for epilepsy and neuroinflammation-related disorders, with ACOD1 as a potential target for CA’s anti-neuroinflammatory effects.

**Figure legends.**

## Electronic supplementary material

Below is the link to the electronic supplementary material.

Supplementary Material 1

Supplementary Material 2

Supplementary Material 3

Supplementary Material 4

## Data Availability

Data is provided within the manuscript or supplementary information files.

## Abbreviations

ABPP: Activity-based protein profiling
CA: Caffeic acid
CNS: Central nervous system
CA-P: Caffeic acid probe
BBB: Blood-brain barrier
LPS: Lipopolysaccharide
ACOD1: Aconitate decarboxylase 1
IL-1: Interleukin-1
IL-6: Interleukin-6
TNF-α: Tumor necrosis factor alpha
PTZ: Pentylenetetrazol
CETSA: Cellular thermal shift assay
LC.MS/MS: Liquid chromatography/tandem mass spectrometry
Elisa: Enzyme-linked immune sorbent assay
