# Supplementary material for: Activity-based chemical proteomics reveals caffeic acid ameliorates pentylenetetrazol-induced seizures by covalently targeting aconitate decarboxylase 1
Source: Cell Commun Signal. 2025 Feb 3;23:62. doi: 10.1186/s12964-024-01739-y (PMC11792687; doi:10.1186/s12964-024-01739-y)
Supplement: Supplementary file 1 — Supplementary Material 1 [file 12964_2024_1739_MOESM1_ESM.docx]

**Activity-based chemical proteomics reveals caffeic acid ameliorates pentylenetetrazol-induced seizures by covalently targeting aconitate decarboxylase 1**

Guanjun Li^#1^, Ling Huang^#1^, Di Gu^#2^ Peili Wang^#3^, Letai Yi^#4^, Wenhua Kuang^1^, Ying Zhang^5^, Junzhe Zhang^5^, Dandan Liu^5^, Qiaoli Shi^5^, Huan Tang^5^, Jichao Sun^1^*, Guohua Zeng^2^*, Xin Peng^6^*, Jigang Wang^1,2, 5, 7^*

^1^ Department of Urology, Shenzhen Clinical Research Centre for Geriatrics, Shenzhen People's Hospital; The First Affiliated Hospital, Southern University of Science and Technology, Shenzhen 518020, Guangdong, China.

^2^ Guangdong Key Laboratory of Urology, The First Affiliated Hospital of Guangzhou Medical University, Guangzhou, 510230, Guangdong, China.

^3^ National Clinical Research Center for Chinese Medicine Cardiology, Xiyuan Hospital, China Academy of Chinese Medical Sciences, Beijing, China.

^4^ Inner Mongolia Medical University, Hohhot 010000, Inner Mongolia, China.

^5^ State Key Laboratory for Quality Ensurance and Sustainable Use of Dao-di Herbs, Artemisinin Research Center, and Institute of Chinese Materia Medica, China Academy of Chinese Medical Sciences, Beijing 100700, China.

^6^ Ningbo Municipal Hospital of TCM, Affiliated Hospital of Zhejiang Chinese Medical University, Ningbo 315000, Zhejiang, China.

^7^ State Key Laboratory of Antiviral Drugs, School of Pharmacy, Henan University, Kaifeng 475004, China.

# Guanjun Li, Ling Huang, Di Gu, Peili Wang and Letai Yi contributed equally to this work.

* Corresponding Authors:

Prof. Jigang Wang, E-mail address: jgwang@icmm.ac.cn

Prof. Xin Peng, E-mail address: pengx@nit.zju.edu.cn

Prof. Guohua Zeng, E-mail address: gzgyzgh@vip.sina.com

Prof. Jichao Sun, E-mail address: sunjichao@mail.sustech.edu.cn

**Table legends
Additional file 2_Table S2.**

Original data of seizure latency and score of each mouse and summary in pentylenetetrazol-induced acute and kindling models of seizures.

**Additional file 3_Table S2.**

Raw data for LC-MS/MS.

**Figure legends.**

**
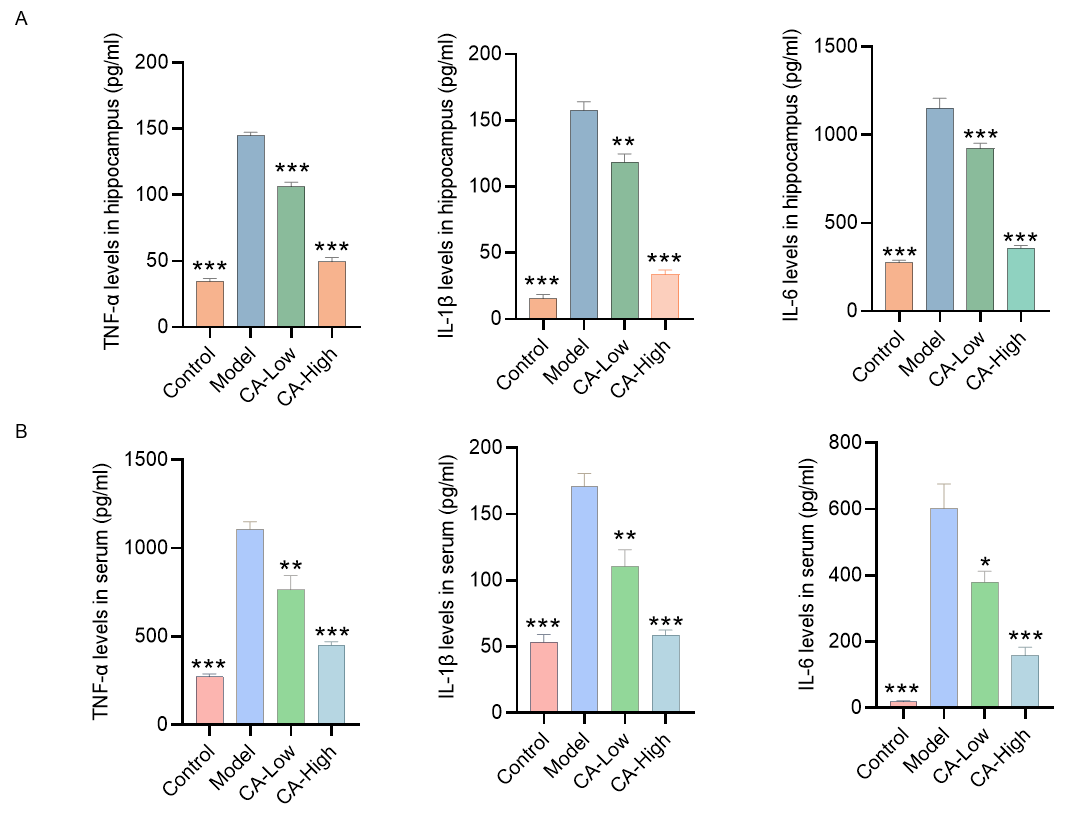
Supplemental Figure 1. The levels of inflammatory factors in mice.** (A) The release of TNF-α, IL-1β as well as IL-6 in the hippocampus of LPS-induced inflammation model mice, n=3. (B) The release of inflammatory factors in serum, n=3. *p<0.05, **p < 0.01, ***p < 0.001 vs model group.

**
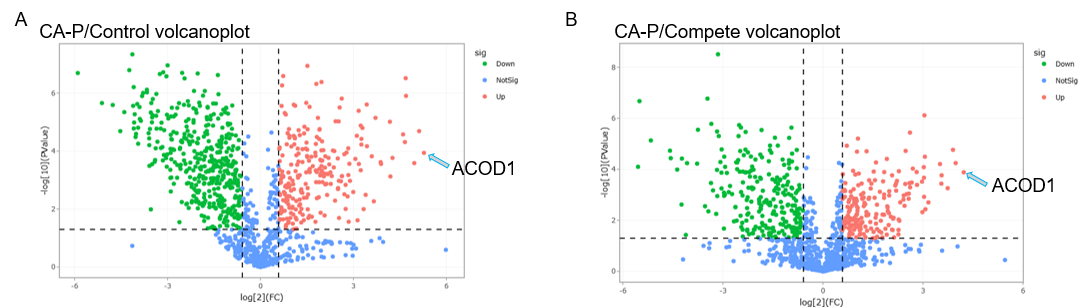
**

**Supplemental Figure 2. Volcanic plot of enrichment proteins.** (A) CA-P/Control volcanic plot of enrichment proteins. (B) CA-P/Compete volcanic plot of enrichment proteins.


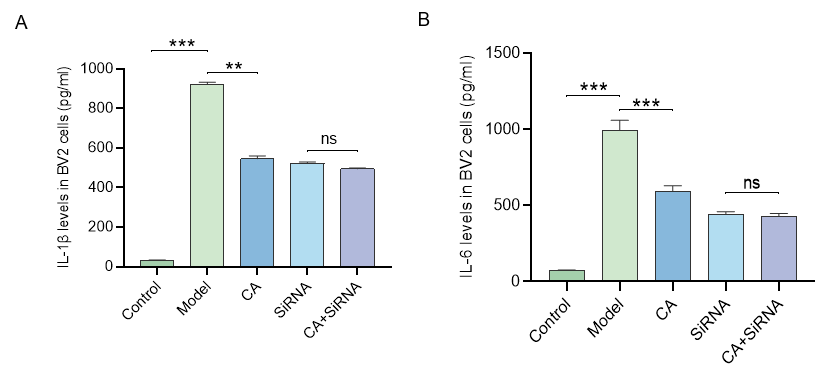


**Supplemental Figure 3. The levels of inflammatory factors in BV2 cells**. (A) The levels of IL-1β in BV2 cells with CA and ACOD1 SiRNA, n=5. (B) The levels of IL-6 in BV2 cells with CA and ACOD1 SiRNA, n=5.
