# Supplementary material for: Activity-based chemical proteomics reveals caffeic acid ameliorates pentylenetetrazol-induced seizures by covalently targeting aconitate decarboxylase 1
Source: Cell Commun Signal. 2025 Feb 3;23:62. doi: 10.1186/s12964-024-01739-y (PMC11792687; doi:10.1186/s12964-024-01739-y)
Supplement: Supplementary file 4 — Supplementary Material 4 [file 12964_2024_1739_MOESM4_ESM.pptx]

## Slide 1
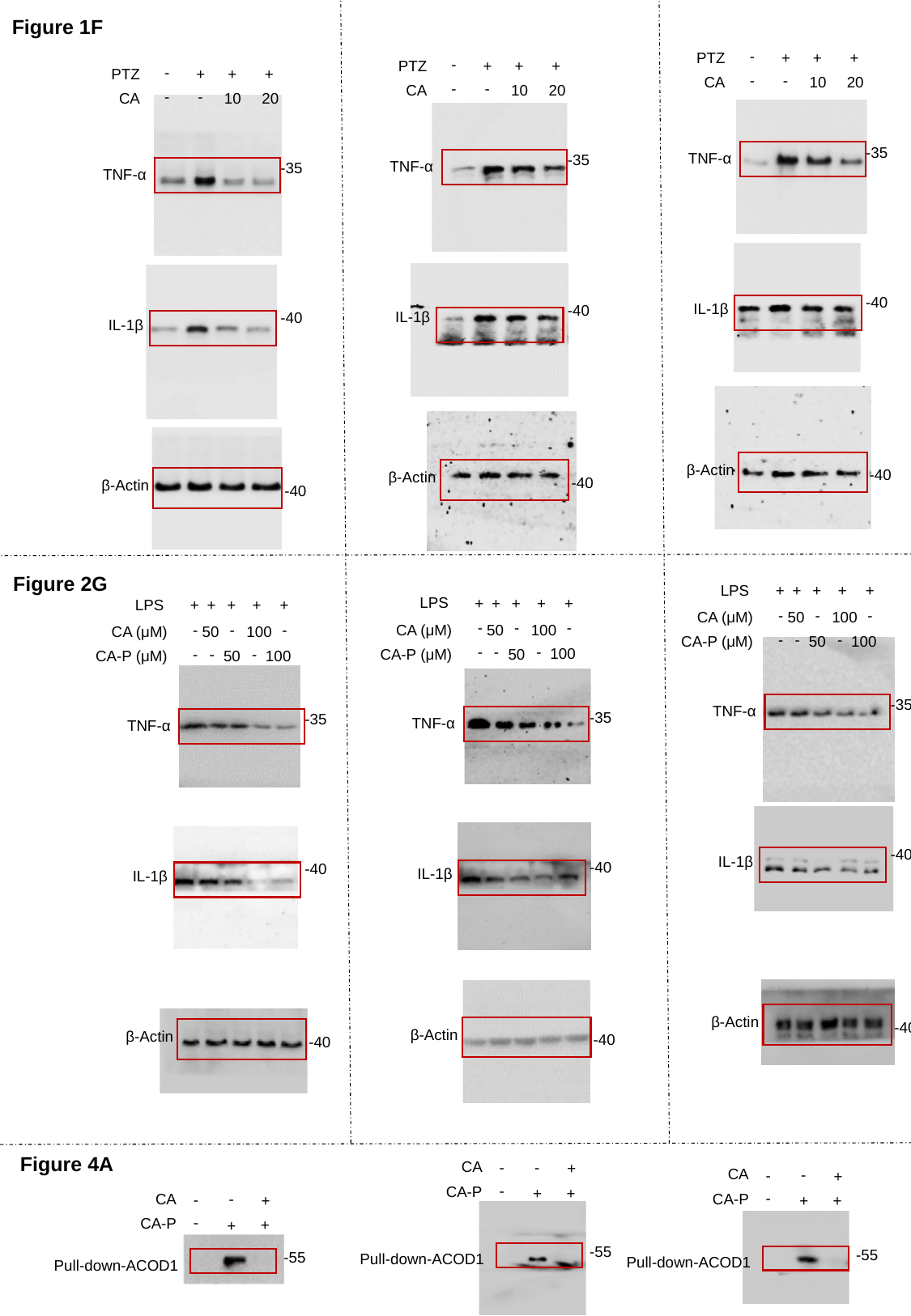

Figure 1F
-
PTZ
+
+
+
-
PTZ
+
+
+
-
PTZ
+
+
+
-
-
CA
10
20
-
-
CA
10
20
-
-
CA
10
20
-35
TNF-α
-35
TNF-α
-35
TNF-α
-40
IL-1β
-40
IL-1β
-40
IL-1β
β-Actin
-40
β-Actin
-40
β-Actin
-40
Figure 2G
LPS
+
+
+
+
+
LPS
+
+
+
+
+
LPS
+
+
+
+
+
-
-
-
CA (μM)
50
100
-
-
-
-
-
CA (μM)
50
100
-
CA (μM)
50
100
-
-
-
100
CA-P (μM)
50
-
-
-
100
CA-P (μM)
50
-
-
-
100
CA-P (μM)
50
-35
TNF-α
-35
-35
TNF-α
TNF-α
-40
IL-1β
-40
-40
IL-1β
IL-1β
β-Actin
-40
β-Actin
β-Actin
-40
-40
Figure 4A
CA
-
-
+
CA
-
-
+
-
CA-P
+
+
-
CA
-
CA-P
-
+
+
+
-
CA-P
+
+
-55
-55
-55
Pull-down-ACOD1
Pull-down-ACOD1
Pull-down-ACOD1

## Slide 2
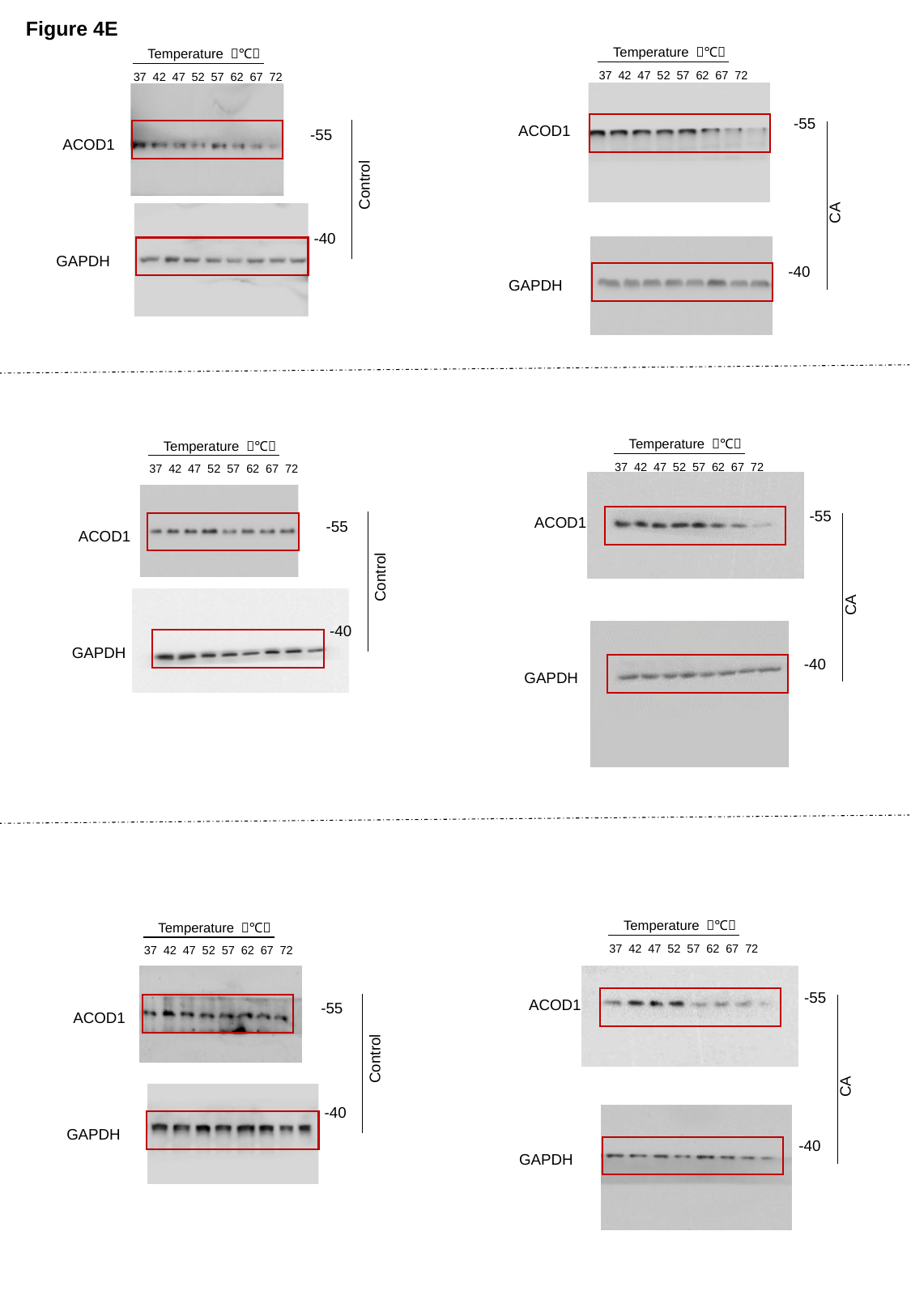

Figure 4E
Temperature （℃）
Temperature （℃）
37 42 47 52 57 62 67 72
37 42 47 52 57 62 67 72
-55
ACOD1
-55
ACOD1
Control
CA
-40
GAPDH
-40
GAPDH
Temperature （℃）
Temperature （℃）
37 42 47 52 57 62 67 72
37 42 47 52 57 62 67 72
-55
ACOD1
-55
ACOD1
Control
CA
-40
GAPDH
-40
GAPDH
Temperature （℃）
Temperature （℃）
37 42 47 52 57 62 67 72
37 42 47 52 57 62 67 72
-55
ACOD1
-55
ACOD1
Control
CA
-40
GAPDH
-40
GAPDH

## Slide 3
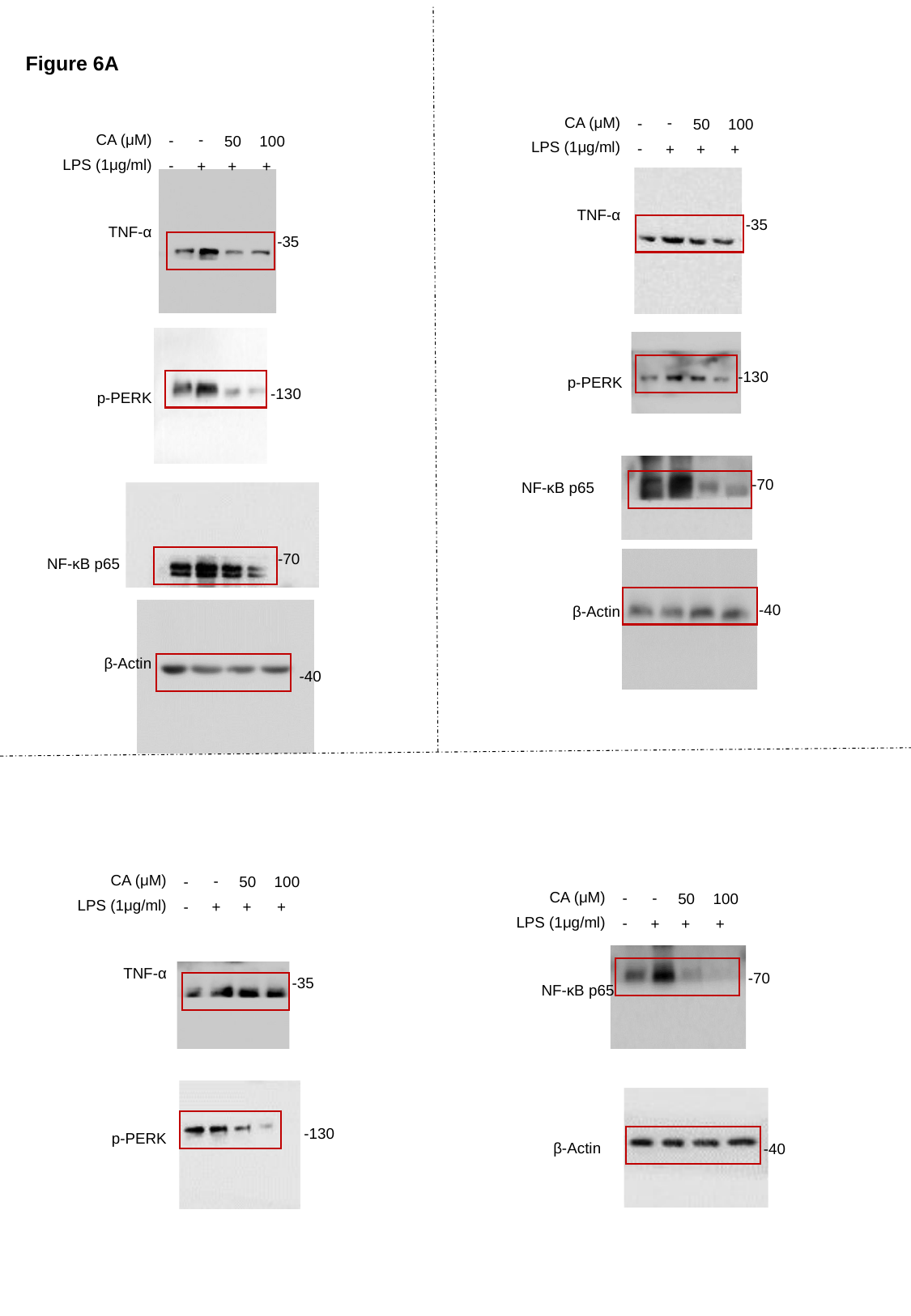

Figure 6A
CA (μM)
-
-
50
100
CA (μM)
-
-
50
100
LPS (1μg/ml)
-
+
+
+
LPS (1μg/ml)
-
+
+
+
TNF-α
-35
TNF-α
-35
-130
p-PERK
-130
p-PERK
-70
NF-κB p65
-70
NF-κB p65
-40
β-Actin
β-Actin
-40
CA (μM)
-
-
50
100
CA (μM)
-
-
50
100
LPS (1μg/ml)
-
+
+
+
LPS (1μg/ml)
-
+
+
+
TNF-α
-70
-35
NF-κB p65
-130
p-PERK
β-Actin
-40

## Slide 4
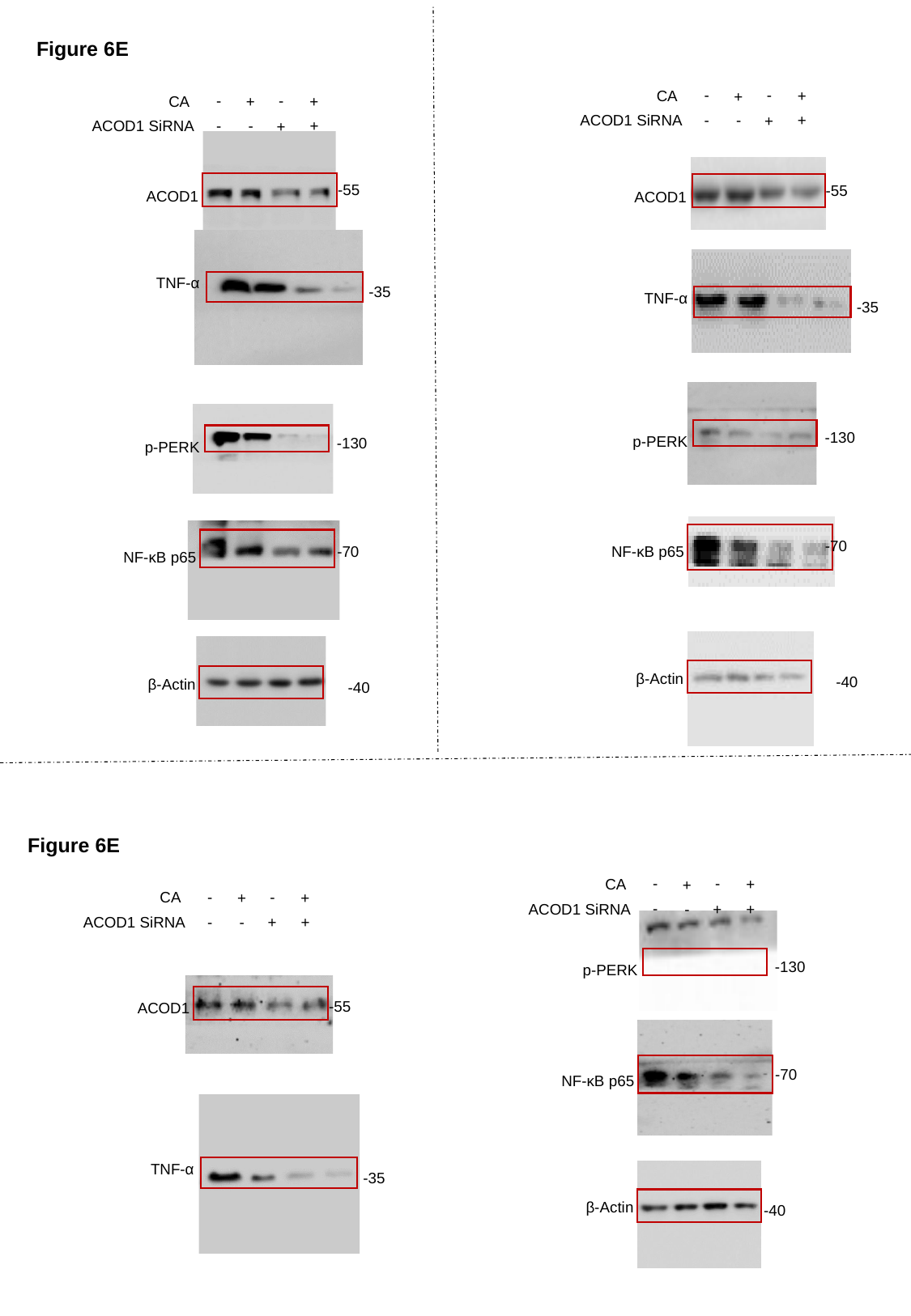

Figure 6E
-
-
CA
+
+
-
-
CA
+
+
-
ACOD1 SiRNA
-
+
+
-
ACOD1 SiRNA
-
+
+
-55
-55
ACOD1
ACOD1
TNF-α
-35
TNF-α
-35
-130
p-PERK
-130
p-PERK
-70
-70
NF-κB p65
NF-κB p65
β-Actin
-40
β-Actin
-40
Figure 6E
-
-
CA
+
+
-
-
CA
+
+
-
ACOD1 SiRNA
-
+
+
-
ACOD1 SiRNA
-
+
+
-130
p-PERK
-55
ACOD1
-70
NF-κB p65
TNF-α
-35
β-Actin
-40
